# A Study on Multimedia Integrated Pre-service Education to Learning Behavior and Competitiveness in Workplace of Employees in Hospitality

**DOI:** 10.3389/fpsyg.2022.817960

**Published:** 2022-07-13

**Authors:** Chih-Hung Pai, Yu-Lan Wang, Yunfeng Shang, Ta-Kuang Hsu

**Affiliations:** ^1^School of Hospitality Management, Zhejiang Yuexiu University, Shaoxing, China; ^2^Teacher Education Institute, Weifang University of Science and Technology, Shandong, China; ^3^Department of Restaurant, Hotel and Institutional Management, Fu Jen Catholic University, New Taipei City, Taiwan

**Keywords:** multimedia, pre-service education, learning behavior, competitiveness in workplace, hospitality, delicacy

## Abstract

The domestic situation of the past few years shows the practices of employees’ unpaid leave and layoffs and the constant drain on capital, talent, and technologies in hospitality. Owners expect to reduce the losses to as low as possible by saving on human costs. Nevertheless, in face of such a changing environment, hospitality has to accumulate high-quality human capital through systematic investment, sensitive development, and continuous learning and growth to discover competitive advantages through the cultivation of human capital. The pre-service education of new employees could accelerate their familiarity with the operations of the company and their understanding of their job role and duties. More importantly, with good planning, it could make employees feel emphasized with and respected with the result of largely changing their thoughts and working habits. Aiming at supervisors and employees in hospitality in Zhejiang as the research objects, a total of 420 copies of our questionnaire are distributed, and 357 valid copies were retrieved, with a retrieval rate of 85%. According to the results to propose discussions, it is expected to generate systematic and proper education methods for the pre-service education in hospitality, promote the education effectiveness, and promote employees’ capability and organizational performance.

## Introduction

Employees are the essential capital for hospitality or any organization where the managers have tried to reinforce human capital. Nonetheless, the domestic situation reveals the practice of unpaid employee leave, layoffs, and the constant drainage of capital, talent, and technologies in hospitality. Owners expect to reduce organizational losses to as low as possible by saving on human costs; however, hospitality should accumulate high-quality human capital through systematic investment, sensitive development, and constant learning and growth and find competitive advantages through the cultivation of human capital. It is the primary issue for managers to transform knowledgeable employees into organizational assets. A talent shortage in an organization would affect the long-term management and even lose good business opportunities. In hospitality, the practice of education is the commonly used and broadly accepted method to reinforce human capital. In order to have the employees learn the skills required for their work, including the knowledge, skills, and behavior necessary to present excellent performance, education aims to have the employees master the competencies required for the work in education courses and apply these abilities to the work (Aarabi et al., 2013).

Under the development trends of globalization, technology, and a knowledge-based economy, hospitality puts a lot of stress on education-related issues in order to enhance the quality of human capital and promote employees’ professional competency. For this reason, hospitality invests lots of resources in education, as pertains to whether pre-service education would develop the business’ educational function and result in positive benefits. As a result, it could promote employees’ competency by understanding and evaluating employees’ competency gaps and designing and planning courses aiming bridging these insufficiencies before education begins. The pre-service education of new employees could accelerate their familiarity with company operations and understanding of their job role and work duties. More importantly, through good planning, it could make employees feel emphasized with and respected, with the result of largely changing their thoughts and working habits. Pre-service education demonstrates the meanings of positions and directions, i.e., assisting new staff in knowing their position and work direction. The content contains the points of the philosophy, objective, organization, policy, environment, and attendance regulations of the company to prevent new employees from reality shock when it comes to executing their actual work (Zumrah et al., 2013). Education is an activity of human resource development. Effective pre-service education could enhance personnel’s ability to do better at work and properly develop to promote work performance. Along with the development wave of the hospitality industry, talent cultivation presents long-term development in the human resource strategy for the hospitality industry. As a result, long-term human resource planning and cultivation are particularly emphasized in the hospitality industry. Meanwhile, the human resource level is promoted from the operational and administrative level to the strategic level to enhance human resource managerial effectiveness and organizational performance. The development, utilization, and management of human resources become critical factors in human and socio-economic development (Paulsen et al., 2015). The possession of talent allows the hospitality industry to present a competitive ability to control the market. In order to retain and acquire talent, human capital and development should be emphasized (Saseen et al., 2017). Multimedia integrated pre-service education to teach behavior and competitiveness in the workplace of employees in hospitality is therefore studied, it is expected to clarify the effect of multimedia integrated orientation training on learning behavior and workplace competitiveness as well as to show systematic and proper education methods for the orientation training in the hospitality industry in order to enhance the educational effectiveness and promote employee ability and organizational performance.

## Literature Review and Hypothesis

Chen et al. (2017) revealed that a hospitality organization could enhance employees’ learning behavior by providing them with pre-service education or encouraging further study. Aiming at staff receiving pre-service education within a year, Kimzey et al. (2019) revealed that those participating in pre-service education presented higher learning behavior after the education and regarded the education as the welfare and being able to enhance the competency. Deci et al. (2017) proposed significant correlations between personality traits and learning behavior that managers in hospitality could arrange different pre-service education according to employees’ personality traits to enhance the learning behavior. Looking at social workers of non-profit organizations in Kaohsiung, Wijma et al. (2018) found positive effects of pre-service education on social workers’ learning behavior.

Lee et al. (2018) regarded remarkably positive correlations between learning behavior and competitiveness in the workplace of employees in the iron and steel industry with high explanatory power and notably positive effects. Looking at employees in Taiwan and China, Mirani et al. (2019) pointed out the positive effects of employees’ learning behavior on the organizational commitment to affect the competitiveness in the workplace. Gholamzadeh et al. (2018) revealed that employees with better outcomes of on-the-job education showed higher learning behavior and better competitiveness in the workplace. Gehrman et al. (2018) discovered that grass-root employees with learning behavior, in finance, would enhance the competitiveness in the workplace at work, i.e., there was a notably positive relationship between learning behavior and competitiveness in the workplace of grass-root employees in finance.

Rickard et al. (2009) stated that employees receiving pre-service education would enhance work efficiency and quality of employees’ work performance and further promote the competitiveness in the workplace. Annear et al. (2017) discovered that most people could learn knowledge and skills in pre-service education for the application to the work as well as to enhance personal ability. Mastel-Smith et al. (2019) found a difference in pre-service education between Taiwanese companies and American companies; however, pre-service education showed positive effects on competitiveness in the workplace in both Taiwanese and American companies. Mavounza et al. (2020) considered that securities specialists, in order to cater to environmental changes, would enhance their competitiveness in the workplace after participating in cross-business pre-service education to show remarkably positive correlations between pre-service education and competitiveness in the workplace.

## Methodology

### Research Framework

**Figure 1:**
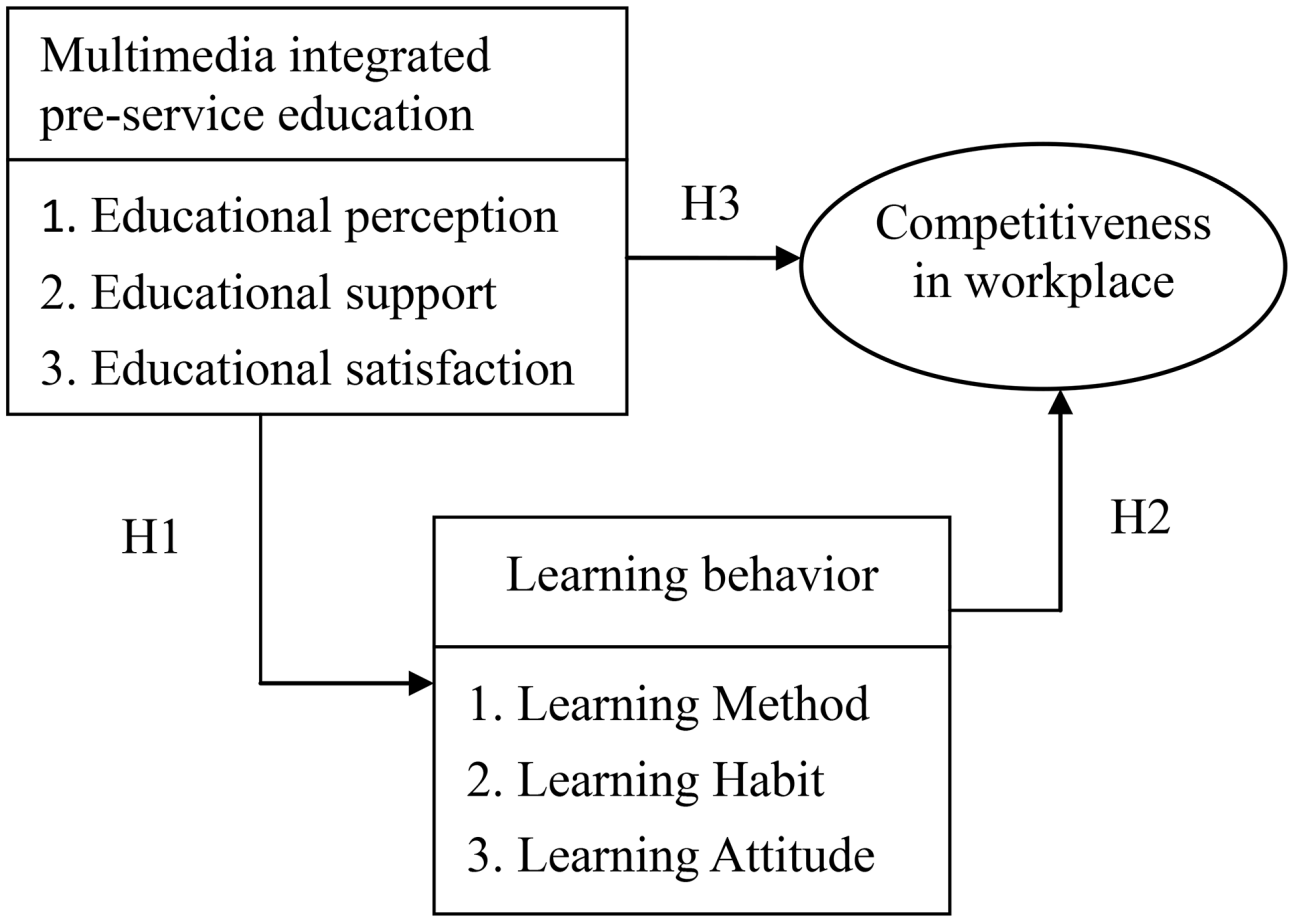
Conceptual framework.

Summing up the above literature review, the conceptual framework (Figure 1) is drawn to discuss the relations between multimedia integrated orientation training, learning behavior, and competitiveness in the workplace.

### Research Questions

According to the above literature review and conceptual framework, the following research issues are acquired.

*H1*: (Hypothesis 1): Multimedia integrated pre-service education shows positive and significant effects on learning behavior.

*H2*: (Hypothesis 2): Learning behavior reveals positive and remarkable effects on competitiveness in the workplace.

*H3*: (Hypothesis 3): Multimedia integrated pre-service education shows positive and notable effects on competitiveness in the workplace.

### Operational Definition

#### Multimedia Integrated Pre-service Education

Referring to the education scale developed by Chan and Leung (2020), three dimensions of educational perception, educational support, and satisfaction with education are contained in this study.

#### Learning Behavior

Referring to the learning behavior scale proposed by Bi et al. (2019), learning behavior is divided into “learning method,” “learning habit,” and “learning attitude” in this study.

#### Competitiveness in Workplace

Referring to the competitiveness in the workplace scale proposed by Wang et al. (2018), competitiveness in the workplace is measured with a single dimension in this study.

### Research Sample and Object

The Zhejiang Food and Beverage Industry Association implements the catering and cooking business policy of “inheritance, development, development, and innovation,” which positively develops the construction of “three fame engineering” (famous restaurants, famous cooks, and famous dishes), organizes provincial and municipal cooking technology competitions in a planned way, participates in national cooking technology competitions and world Chinese cooking competitions, and develops activities of Zhejiang cuisine skills training and exchanges inside and outside the province to develop the important function for the prosperity of hospitality market and the development of Zhejiang dishes. Aiming at supervisors and employees in hospitality in Zhejiang, a total of 420 copies of our questionnaire were distributed, and 357 valid copies retrieved, with a retrieval rate of 85%.

### Reliability and Validity Test

Confirmatory Factor Analysis (CFA) is an important part of SEM that the measurement model of the structural model should be tested before the two-stage model modification. When the model fit is acceptable, the second step SEM model evaluation is preceded. The CFA reveals the factor loadings of dimensions in 0.60~0.90, the composite reliability in 0.70~0.90, and the average variance extracted in 0.60~0.80, conforming to the standards of (1) factor loadings > 0.5, (2) composite reliability > 0.6, and (3) average variance extracted > 0.5. The dimensions, therefore, present convergent validity.

## Results

### Structural Model Analysis

The structural model analysis contains the goodness-of-fit analysis of the research model and the explanatory power of the overall research model. In this case, seven numerical indices are used for testing the goodness-of-fit of the overall model, including the chi-square (*χ*^2^) test, *χ*^2^-degree of freedom ratio, the goodness of fit index, adjusted goodness of fit index, root-mean-square error, comparative fit index, comparative hypothesis model, and chi-square of the independent model. The overall results are organized in Table 1.

**Table 1:** Research model fit analysis.

Fit indices	Allowable range	This research model	Model fit judgment
*χ*^2^ (Chi-square)	The smaller the better	17.68	
*χ*^2^-degree of freedom ratio	<3	1.94	Match
GFI	>0.9	0.96	Match
AGFI	>0.8	0.83	Match
RMSEA	<0.08	0.05	Match
CFI	>0.9	0.92	Match
NFI	>0.9	0.91	Match

From the above indices, when using the *χ*^2^-degree of freedom ratio for testing the model, the ratio is better smaller; this research model shows the *χ*^2^-degree of freedom ratio <3 (1.94). GFI and AGFI are better close to 1, have no absolute standards to judge the fit, and GFI >0.9 and AGFI >0.8 are acceptable; this research model reveals GFI and AGFI 0.96 and 0.83, respectively. RMSEA being in 0.05–0.08 stands for a good model and reasonable fit; this research model shows RMSEA of 0.05. The allowable standard of CFI is >0.9, and this research model shows CFI as 0.92. NFI should be at least higher than 0.9, and this research model reveals the NFI is 0.91. Overall speaking, the goodness-of-fit indices conform to the standards, revealing the model to be acceptable. The research data therefore could explain the actual observed data.

From the above-mentioned overall model fit indices, the model structured in this study and the observed data present a favorable fit, revealing that the theoretical model could fully explain the observed data. Consequently, after the model fit test, the correlation coefficient and coefficient estimate of multimedia integrated pre-service education to learning behavior and competitiveness in the workplace could be understood. The research hypothesis test results are shown in Table 3.

The research data are organized in Table 2. The overall model analysis results show that three dimensions of multimedia integrated pre-service education (educational perception, educational support, and satisfaction with education) could significantly explain multimedia integrated pre-service education (*t* > 1.96, *p* < 0.05), three dimensions of learning behavior (learning method, learning habit, and learning attitude) could remarkably explain learning behavior (*t* > 1.96, *p* < 0.05), and competitiveness in the workplace presents the notable explanation (*t* > 1.96, *p* < 0.05). The overall model appears to present a good preliminary fit.

**Table 2:** Overall linear structural model analysis result.

Evaluation item	Parameter/evaluation standard		Result
Preliminary fit	Multimedia integrated pre-service education	Educational perception	0.70^*^
		Educational support	0.67^*^
		Satisfaction with education	0.75^**^
	Learning behavior	Learning method	0.69^*^
		Learning habit	0.73^**^
		Learning attitude	0.71^**^
Internal fit	Multimedia integrated pre-service education → learning behavior		0.81^***^
	Learning behavior → competitiveness in workplace		0.86^***^
	Multimedia integrated pre-service education → competitiveness in workplace		0.83^***^

^*^p < 0.05, ^**^p < 0.01, ^***^p < 0.001.

Regarding the internal fit, multimedia integrated pre-service education shows positive and significant correlations with learning behavior (0.81, *p* < 0.01), learning behavior reveals positive and remarkable correlations with competitiveness in the workplace (0.86, *p* < 0.01), and multimedia integrated pre-service education shows positive and notable correlations with competitiveness in workplace (0.83, *p* < 0.01). H1, H2, and H3 are therefore supported. The research hypothesis test results are shown in Table 3.

**Table 3:** Hypothesis test.

Research hypothesis	C	Empirical result	*p*	Result
H1	+	0.81	<0.01	Supported
H2	+	0.86	<0.01	Supported
H3	+	0.83	<0.01	Supported

From the research results, multimedia integrated orientation training presents positive and significant effects on learning behavior (0.81, *p* < 0.01), showing that H1 is supported. Apparently, in order to cope with environmental changes, the hospitality industry applies multimedia integrated orientation training to promote employees’ learning behavior, i.e., remarkably positive effects of multimedia integrated orientation training were found on learning behavior. Learning behavior shows positive and remarkable effects on workplace competitiveness (0.86, *p* < 0.01), meaning that H2 is supported. In this case, when the hospitality industry is able to provide relevant multimedia integrated orientation training courses, the employees feel that the organization is taking care of and is responsible for them. Consequently, employees would present more rigorous learning behavior for the affirmation of self-capability to further promote workplace competitiveness. Finally, multimedia integrated orientation training reveals positive and notable effects on workplace competitiveness (0.83, *p* < 0.01), showing that H3 is supported. Apparently, in a time with multiple knowledge-based economies, the hospitality industry promotes multimedia integrated orientation training to enhance employees’ professional knowledge and skills or encourages employees to cultivate learning behavior in order to promote workplace competitiveness.

## Discussion

Under the premise of satisfying customer requirements, hospitality should present sufficient and excellent professional technology and develop selectively. The enhancement of customer satisfaction should result in a repeat visit and even a recommendation to friends. On the contrary, dissatisfied customers might not visit again and might urge others not to visit. Since professional technology and sensitive development are so important, multimedia integrated pre-service education should be particularly emphasized. With existing bases, enhancing personal professional technology would promote the competitiveness in the workplace. Businesses in the hospitality industry are suggested to positively and effectively establish a training program and system for trainers as well as understand orientation-training related instructional methods and communication skills with orientation training lecturers to further promote the teaching quality of internal orientation training (Hyndman and Pill, 2017). As a result, experts and scholars could be invited to participate in the practical lectures and professional technology demonstration of multimedia integrated pre-service education to instruct and correct the employees’ professional technology and sensitivity development and promote competitiveness. In order to have their personal ability be affirmed, the employees in hospitality would make efforts and learn professional technology and related professional knowledge from multimedia integrated pre-service education. In this case, preceding learning behavior for the application to customer needs and enhancing customer satisfaction to promote work performance and competitiveness in the workplace would enhance the career development of the employees in hospitality as well as the coherence. Employees in the hospitality industry should regularly check and promote hardware facilities and attempt to apply interactive multimedia interactive teaching and materials or other relevant educational equipment or teaching media to stimulate the employees’ learning willingness and interests as well as achieve the training objective and further enhance training effectiveness (Appleby et al., 2016). For instance, they can utilize audiovisual teaching for self-learning, i.e., using electronic equipment of films, video files, and recorders for delivering teaching content. It is suggested that the hospitality industry, in addition to stressing employees’ educational training, should list constant expenses for purchasing educational training-related equipment to promote educational effectiveness (Cullen, 2016).

## Conclusion

The research results reveal positive effects of multimedia integrated orientation training on learning behavior and workplace competitiveness of employees in the hospitality industry. Employees could learn better and utilize more professional technology in pre-service education; once the professional technology is promoted, the learning behavior would be enhanced. The research results reveal the greater the learning behavior of employees in hospitality, the larger the competitiveness in the workplace. Employees being willing to participate in learning behavior and further make extra efforts for the learning behavior would enhance competitiveness in the workplace. The research results reveal that employees in hospitality attending more multimedia integrated pre-service education would present stronger competitiveness in the workplace. Hospitality requires professional technology and skills that the employees have to regularly participate in relevant technology study or on-the-job education courses for discussion and exchange with each other. It could enhance professional skills and further promote customer satisfaction and loyalty to help the promotion of competitiveness in the workplace. Roetert and Jefferies (2014) mentioned that there was not “the best” training method, but merely “the most suitable” method. Any training courses to achieve the training objective with multiple training methods would show higher effectiveness than those with merely one method. With similar research results to this study, French et al. (2015) revealed that multimedia integrated orientation training presented better assistance to the effectiveness of employees in the hospitality industry. Accordingly, the orientation training objects and the required skills, knowledge, or attitude should be carefully considered in the construction of orientation training content and planning of orientation training programs before the practice of orientation training courses. Proper orientation training methods are then selected with appropriate orientation training materials and relevant teaching to achieve the expected orientation training effectiveness (Kilcommins and Spain, 2016).

## Data Availability Statement

The original contributions presented in the study are included in the article/supplementary material, further inquiries can be directed to the corresponding author.

## Ethics Statement

This study was reviewed and approved by the ethics committee of the Zhejiang Yuexiu University. Written informed consent was obtained from all participants for their participation in this study.

## Author Contributions

C-HP and Y-LW: collected and analyzed the data and drafted the manuscript. YS: designed the research protocol and contributed to the literature review. T-KH: reviewed the manuscript and revised the manuscript. All authors revised and approved the submitted version of the manuscript.

## Conflict of Interest

The authors declare that the research was conducted in the absence of any commercial or financial relationships that could be construed as potential conflicts of interest.

## Publisher’s Note

All claims expressed in this article are solely those of the authors and do not necessarily represent those of their affiliated organizations, or those of the publisher, the editors and the reviewers. Any product that may be evaluated in this article, or claim that may be made by its manufacturer, is not guaranteed or endorsed by the publisher.

## Funding

This work was supported by 2020 Zhejiang Social Science Project, Zhejiang Province, China (Grant Number: 20XXJC04ZD).
